# Word-of-mouth among blood service employees who also donate blood: a qualitative investigation of advantages and challenges for dual-role donors

**DOI:** 10.1186/s12913-024-11181-y

**Published:** 2024-06-14

**Authors:** Kathleen Chell, Michael Polonsky, Barbara Masser, Melissa K. Hyde

**Affiliations:** 1 Australian Red Cross Lifeblood, Queensland, Australia; 2 The University of Queensland, Queensland, Australia; 3 Deakin University, Victoria, Australia

**Keywords:** Blood donation, Blood service employees, Word-of-mouth

## Abstract

**Background:**

Despite word-of-mouth (WOM) and electronic WOM (eWOM) influencing people’s willingness to donate blood, no research has explored this behavior among blood service employees who are also donors. This underexplored segment is highly important, as they are generally committed to both the organization and the cause and are likely more informed on the topic of blood donation than the average donor.

**Methods:**

This study comprised six online focus groups with 26 Australian Red Cross Lifeblood employees who are also donors. Questions covered a range of blood donation and WOM topics, including when they became blood donors, if they had engaged in WOM about blood donation, what they had talked about and with whom, and what were audience reactions. Thematic analysis was then used to explore how responses related to the employees’ motivations, opportunities, and abilities to engage in WOM and eWOM about blood donation.

**Results:**

While most employee-donors saw alignment in their employee and donor roles, advocating for blood donation was not considered a necessary part of either role. Educating others about blood donation was a common goal of employee-donor WOM and eWOM, and almost all employees engaged in reactive WOM, triggered by events (e.g., recent donations) or questions about their work. Employee-donors in donor-facing roles (e.g., communications and collections staff) felt more aware of the importance of encouraging others to donate blood and were also more likely to be proactive in their WOM activity. Along with these perceived advantages of having a dual role, employee-donors also identified some disadvantages, such as unrealistic expertise expectations and negative audience responses that can be difficult to navigate.

**Conclusions:**

Being an employee-donor is a double-edged sword. For example, increased opportunities to talk about blood donation and access to more information can be offset by having to respond to more challenging questions/comments and expectations, while appropriately representing their employer. More research is needed among those in employee-donor roles within the healthcare and/or non-profit sectors, to determine whether these are issues faced more broadly, and how those in dual roles can be most effectively supported to engage in positive WOM and eWOM.

**Supplementary Information:**

The online version contains supplementary material available at 10.1186/s12913-024-11181-y.

## Background

Word-of-mouth (WOM) and electronic WOM (eWOM) are often described as informal, interpersonal exchanges of information about a product or service [1, 2], and are regarded as more reliable and influential in consumer decision-making than commercial information sources [3, 4], including in health related contexts [5]. Individuals that engage in positive WOM and eWOM act as critical marketing advocates for organizations [6]. Such advocates do more than simply promote key elements, they have even been found to defend the organizations when they encounter negative feedback or comments about the organization [7].

While traditionally explored in a commercial context, WOM and eWOM are also important in terms of promoting health [8] and other prosocial behaviors [9], such as blood donation [5]. This is because such behaviors are intangible, complex, and of a high-risk to low-benefit nature [10]. In the context of this study, positive WOM and eWOM about blood donation from existing donors can increase awareness, positive feelings, commitment, and intentions toward donating blood [2, 5, 8, 11–14], and have been deemed necessary to increase its salience and translation into a normative behavior [15, 16]. The World Health Organization and the International Federation of Red Cross and Red Crescent Societies emphasized the need for “*improved public awareness of the importance of blood donation as a social norm* (p2)” to ensure a sustainable amount of safe blood [17]. Yet despite evidence that blood donors are generally willing to talk about blood donation [2], many lack the motivation, opportunity, and/or (cap)ability to do so [18, 19]. Models such as the Motivation, Opportunity, and Ability (MOA) framework [20] or the related Capabilities, Opportunity, and Motivation – Behavior (COM-B) model [21] have sought to understand how these components interact. In blood donation, health or other prosocial contexts, the impact of there being a lack of motivation, lack of opportunity and/or a lack of (cap)ability is not often discussed. In a commercial context, WOM is often reactively triggered by situational and social factors [22], such as customers being asked for advice [20, 23, 24]. However, asking for advice may not naturally occur for health and prosocial behaviors, including blood donation.

Extensive research has investigated preconditions for WOM and eWOM in prosocial contexts [1, 8, 22, 25]. Some of this research has contended that satisfied customers, health service patients, and blood donors, are more willing to share positive WOM [2, 21] and are also more engaged with the organizations that deliver these positive experiences [26]. In line with this, a positive (vs. negative) blood donation experience has been shown to improve (reduce) donor retention (loss) and the likelihood (reduction) of positive WOM and eWOM [27, 28]. Guglielmetti et al. (2021) found that as donation and WOM intentions increased, individual’s perceived need to raise community awareness of blood donation also increased, in either in-person or online communication. WOM intentions also increased when people were satisfied with service quality [5]. Martin et al. (2019) similarly identified that most blood donor WOM recommendations were dependent on whether there were ‘friendly employees’ at the blood service (97%), the blood donation was a positive experience (97%), there were short waiting times (97%), and detailed information was available (96%) [29]. Furthermore, the emotional value gained from the donation experience [10, 30], social norms around donating blood [31, 32], as well as self-efficacy toward recruiting other donors, perceived recruitment responsibility, and past donation behaviors and moral norms [33] have all been found to enhance positive WOM and eWOM among blood donors.

While WOM and eWOM have generally been explored among consumers and donors, other research has examined employees engaging in WOM about their employer and their employers’ activities. In this research, employee WOM has been referred to as ‘employee advocacy’ [34] or being an ‘employee brand ambassador’ [35]. Although research has examined how to motivate employees to positively represent their organization via WOM and eWOM [36, 37], other studies highlight that employees often do not want to take on an ambassadorial role [38]. This ambassadorial role has also been considered at the organizational level, with many organizations establishing rules and guidelines about what employees can say and how employees undertake WOM and eWOM [37, 39, 40], especially negative WOM [41].

There are many situations where employees are also consumers of the organization’s goods and services. In the context of prosocial services such as blood donation, employees are also often donors whose values are aligned with their employer’s cause [41]. It was therefore anticipated in this study that employee-donors’ positive views about blood donation would be reinforced by their positive attitudes towards their employer. That is, they work for the organization and further support its mission by giving blood themselves.

Such employees are typically considered as one aspect of the overall service process, in terms of shaping donor experiences that drive donors’ positive or negative WOM [5]. Yet employee-donors are also a potential source of positive WOM or eWOM for their organization’s prosocial services, including blood donation. There has been limited discussion about employee-customer or employee-donor WOM or eWOM activity, despite them being a uniquely informed cohort within health and prosocial contexts. In terms of blood donations, past research has shown that having professional health-related roles with additional knowledge and understanding of the process of blood donation can deliver positive endorsements that improve intentions [42, 43]. In line with this, Pauli, Martin, and Greiling (2023, p142) suggested that “*healthcare workers … could act as ambassadors that spread their positive impressions (e.g. WOM) of the healthcare service among their clusters of family, friends and acquaintances*” [8].

This research seeks to deepen the understanding of WOM and eWOM by investigating employees with dual roles in health and/or prosocial services – specifically those that work for blood services and donate blood. Qualitative methods were used to explore the current WOM practice as well as motivations, opportunities, and abilities for WOM and eWOM about blood donation among Australian Red Cross Lifeblood (Lifeblood) employee-donors. An assessment was also made of whether the experiences of these dual-role donors differed, based on whether their role was donor-facing (e.g., donor center, marketing, call center) or organization-facing (e.g., manufacturing, R&D, finance). This study drew on the MOA framework [44], which asserts that WOM and eWOM about blood donation not only depend on an individual’s motivation to talk positively about donating blood, but also on their opportunity and ability to do so [19]. After further discussing the research method below, this study’s results and implications for practice and theory are provided.

## Method

### Design

Given the limited research on how employee-donors perceive their role in and sharing of WOM, qualitative methods were applied in this study, to understand their lived experiences [45]. Qualitative approaches have often been used within the heath service domain to understand behavior [46], including in the context of blood donation WOM [5].

### Setting

Lifeblood is the sole organization responsible for blood collection in Australia and employs over 3,500 people [47]. Across Australia there are 78 blood donor centers, 18 mobile centers and 6 pop-up centers. There are also national and state offices, where processing and operational activities are undertaken, where large numbers of employees work, but do not have collection facilities.

Life blood has a range of media and social media policies. All Lifeblood staff complete an online training module about social media use in general (e.g., privacy and security) and on content that should (not) be posted.

This study comprised six online focus groups with a convenience sample of 26 participants who were Lifeblood employees who worked in a range of functional areas across Australia and had previously donated blood in Australia (see Table 1). This sample size is comparable to that observed in other WOM research in blood donation [5]. After receiving ethics approval from the Lifeblood Human Research Ethics Committee (ethics reference number 2021#10), employee-donors were recruited via internal electronic communications (e.g., staff newsletter, intranet, and executive email updates). This was necessary as Lifeblood does not record whether staff are donors or not, and the donor status of an employee is generally not known to other employees unless that the donor chooses to disclose this (either in conversation or by presenting to a donor center where colleagues known to them work). Communications advertising the study specified the goal of the project – that is of discussing WOM with employees who donated, and participants were invited to contact the research team through completing a short demographic information survey to indicate their interest. These surveys also included questions on respondents’ age, gender, country of birth, donation history, donation recency, employee tenure and work division (see Supplemental Materials).

Employee-donors’ work division was considered to categorize staff in terms of whether their role involved regular contact with donors and potential donors (i.e., Donor-Facing [D-F], such as working in a donor center, marketing team or call center) or was more organization-facing (O-F) (e.g., working in manufacturing, R&D, or finance), to explore whether this also influenced WOM. The survey also listed alternative meeting times to hold the focus group session and maximize opportunity for participation. Signed consent forms were collected by email prior to data collection. All focus groups occurred during standard office hours (9am to 5pm on weekdays), and so employee-donors were not compensated for their time (even though some did participate outside of their normal working hours). In organizing the focus group eight volunteers were not able to attend at the times available.

### Data collection

The focus groups were facilitated via Microsoft Teams, an online meeting and collaboration tool regularly used by Lifeblood staff, with each running for 1.5 to 2 hours and with participants in a location of their choosing. Each participant took part in one of the 6 focus group sessions. The discussions were moderated by a male and female member of the research team (MP and KC), who both have PhDs and have previously published qualitative works. One member of the moderation team was employed as a research fellow at Lifeblood and the other in an academic role at an Australian university. No other non-researchers or participants were in attendance. The moderators and broader research team have all undertaken research with Lifeblood for many years, but not all are donors.

The focus groups were video-recorded and transcribed by an external company with which Lifeblood has a confidentiality agreement in place. Participants were not given opportunities to review the materials. The questions covered whether employee-donors had talked about blood donation, what they had discussed, and how, when, where, and with whom this occurred, as well as WOM audience reactions (see Supplementary Materials). Within the focus groups, the moderators acted to ensure that no one respondent dominated discussions. Specifically, for each question, all respondents were given the opportunity to respond, with moderators asking “what do you think respondent X” when necessary, to ensure all respondent views were expressed and incorporated. In addition, the moderators asked people whether they disagree or had alternative views.

The members of the research team had no formal relationship with participants and, reflecting the size and geographical spread of the organization, were unknown to the moderators and broader research team. It is, however, possible that those employees who volunteered may have known of the researchers (e.g., through attending business-wide seminars etc.).

### Data analysis

Following data collection, each participant was given a code (i.e., respondent 1 - respondent 26) for the purpose of analysis and to maintain anonymity. The focus group transcripts were thematically coded allowing for a “*theoretically flexible approach to analyzing qualitative data* (p77)” [48] and qualitative data to be rigorously analyzed to deliver credible results [49]. Thematic analysis has been used in other studies that have explored blood donations and WOM/eWOM [32] and is recognized as effective in exploring individuals’ lived experiences [50]. The initial codes were developed by one member of the research team (MH) and then cross validated and refined by two other members (MP and KC). The codes were agreed on by the three coders. Themes were assessed against the literature on WOM, but, as no studies have examined employee donors, new themes arose. While there was a higher degree of agreement (saturation) among participants, alternative views were identified in some instances, and these are also discussed. Once manual coding was complete, the initial background survey was referred to. This allowed the researchers to identify whether employee-donors’ roles were donor-facing (D-F) or organization-facing (O-F).

**Table 1:** Participant demographics

	Donor-Facing (D-F) role (roles that involve contact with donors, e.g., donor centers, marketing)				Organization-Facing (O-F) role (roles that are operationally focused, e.g., manufacturing, *R*&D, finance)				Total
*Age in years*	*All*	*18–29*	*30–49*	*50+*	*All*	*18–29*	*30–49*	*50+*	*All*
Female	10	5	4	1	7	3	1	3	*17*
Male	5	1	4	0	4	1	2	1	*9*
Born in Australia	11	4	6	1	9	4	3	2	*20*
Born in another country	4	2	2	0	2	0	0	2	*6*
≤ 6 months since last donation	11	4	6	1	9	3	2	4	*20*
> 6 months since last donation	4	2	2	0	2	1	1	0	*6*
4–10 total donations	3	2	1	0	2	1	1	0	*5*
> 10 total donations	12	4	7	1	9	3	2	4	*21*
< 5 years Lifeblood employee	10	5	4	1	7	4	1	2	*17*
≥ 5 years Lifeblood employee	5	1	4	0	4	0	2	2	*9*
*Total*	*15*	*6*	*8*	*1*	*11*	*4*	*3*	*4*	*26*

## Results

### Sample composition

The sample is designed to include the views of employees of Lifeblood who are donors. While qualitative research is not designed to be assessed using statistical techniques, it is important to determine whether participants are broadly reflective of the population. Two populations are relevant here – the broader donor panel and Lifeblood employees^1^. Consistent with the majority of Lifeblood employees (77%) but not the donor panel (50%), most of our sample (64%) identified as female. Further, and consistent with the donor panel (74%) most of our sample (77%) were born in Australia. However, different to both the donor panel and Lifeblood employees in general, our sample was, on average, younger. Specifically, 38% of our sample were aged 18–29, compared to 20% of the donor panel and 24.4% of Lifeblood employees (aged 25 and under). This may reflect the fact that we recruited employees to discuss WOM and eWOM, with eWOM often highly salient to younger people and donors.

These results are focused on four themes guided by the MOA framework. The first is whether employees donated before or after starting employment at Lifeblood, in terms of how alignment in their dual role occurred. The second explores what motivates D-F and O-F employee-donors to talk about blood donation, and how each role drives WOM/eWOM. The last two themes explore the advantages and challenges of WOM and eWOM, by considering both employee-donor opportunity and ability to talk about blood donation and respond to questions, and whether this differs from other research on non-employee donors. Each theme investigates whether motivation, opportunity, and ability to talk about blood donation differs (or not) between employees in D-F and O-F roles.

### Employment and blood donation

Most participants (n = 18, 69%) were blood donors before they joined Lifeblood as employees. Even though they were not asked why they wanted to work at Lifeblood, four mentioned their desire to work there because of their commitment to blood donation:

*“I initially started donating quite a few years ago and then after a few years of donating I asked if they needed volunteers, so then I started coming every second Saturday to volunteer at my donor center. [I] did that for a few years and really, really liked – wanted to try and work with the company.” (Respondent 8, O-F)*.

Two of them viewed working at Lifeblood as an opportunity to increase the impact they could have (i.e., helping to facilitate other people donating) in addition to their own donations:

*“The fact that I was a blood donor was what made me really want to work for Lifeblood … I was excited to join the team and know the extra impact I could make on people’s lives. That I was only – I was limited every two weeks or whatever it was, to making that difference, but now I get to do it every day.” (Respondent 1, O-F)*.

The above response highlights the alignment between donating blood and organizational values, which is common in non-profits [45]. In the context of Lifeblood, this relates to employees focused on helping others by donating while also expanding their role and influencing others to donate.

Among the eight participants that did not donate before working at Lifeblood, all felt inspired to do so because of their employment. That is, the organizational values of supporting blood donation were transferred to these employees. In the focus groups, these participants did not indicate a sense of obligation or pressure to donate, but rather that their employment at Lifeblood had created an opportunity that they had not previously considered:

*“It [blood donation] just never came across my radar until I started working here.” (Respondent 13, D-F)*.

*“I actually always thought about it, never really got around to doing it, and never realized the importance of it until starting here.” (Respondent 16, D-F)*.

Whereas some employees felt it would be inconsistent or incompatible for them to work at Lifeblood and not donate if they were eligible to do so:

*“I didn’t think it was right for me to apply for a job and not know anything about it, so I went and donated.” (Respondent 20, O-F)*.

*“I thought it was probably poor form to not donate if you work here, and you can so that’s pretty much it, I think.” (Respondent 21, O-F)*.

Such motivation to donate often stems from a need for self-congruency between an action and self-concept [52]. In line with this, some employees felt more authentic when talking about blood donation and encouraging others to donate, as they based this on their lived experience:

“*I think I do it now that I’ve had the experience personally to be able to spread the word a little bit.”* (*Respondent 16, D-F)*.

“*I think when I’m really active in my donations, I’m more willing and I’m more forthcoming when it comes to trying to push others to do it. But then when I’m not doing it as much, I think it’s a bit hypocritical of me to be like ‘You need to go and donate’, and I’m not necessarily donating all the time either*.” (*Respondent 21, O-F*).

Having first-hand experience of the donation process was viewed as particularly beneficial among participants in D-F roles:

“*Now for me, it’s more of an experience to understand how it all works and I can learn as much more as I can … I’ve tried to go around to different donor centers to get a flavor [understanding] of different experiences that I get [at different donation centers].”* (*Respondent 16, D-F*).

“*But it makes it really easy for me to put myself in a donor’s stand-of-view, so don’t make them wait, or treat them as I wanted to be treated as a donor. That really makes me more attentive to the donor*.” (*Respondent 26, D-F*).

### WOM motivation in a dual role

Among the employee-donors there appeared to be a common alignment in values associated with donating blood and their role working for Lifeblood. This corresponded to a greater likelihood of engaging in WOM and eWOM about blood donation. Many participants felt more motivated to talk about blood donation since commencing at Lifeblood:

“*I do now, but it’s really only been since working for Lifeblood that I talk to other people about it, but before that I never did*.” (*Respondent 4, O-F)*.

“*Working there gives me much more incentive to bring it up in the conversation.*” (*Respondent 19, D-F*).

It is unclear whether these participants felt their role as an employee gave them more authority to talk about blood donation or that promoting blood donation via WOM was a broader responsibility, similar to being a brand ambassador [35]. At least one participant did not see themselves as a brand ambassador: “*I don’t think I would personally consider myself a recruiter or see that as part of my role*” (Respondent 19, D-F). Yet this same participant indicated that when they talked about their job, they “*ended up talking about it [blood donation] a lot*”.

There were also some who consciously tried to separate their employee and donor roles when discussing donating blood and Lifeblood, to appear more genuine and appear to be less of a spokesperson. Despite their employee-donor dual role, many participants still considered their WOM about blood donation to be driven from mostly being a blood donor:

“*I definitely talk about it more as a donor, so my experience as a donor, not as a staff member at all.*” (*Respondent 20, O-F*).

“*I feel like I still see myself as a donor, but now I’m working at Lifeblood I feel like I have to be a bit more delicate in how I discuss it, so as not seen as being too pushy to get people to donate.*” (*Respondent 12, O-F*).

“*I just get the response of, ‘You have to say that because you work there’. And I’m, ‘But I’ve been donating for over 10 years now, so it’s not just ‘cause I work there’. That’s just my general response.*” (*Respondent 15, D-F*).

Alternatively, some participants reflected that since working at Lifeblood they were more aware of the importance of donating blood, and the importance of encouraging others to donate, particularly those in D-F roles:

“*Working here has made me realize how much more important than I originally thought it [WOM about blood donation] was. Then it’s just become a thing where it’s like, ‘Dude, it’s important. You’ve got to do this. It really does help people’.*” (*Respondent 5, D-F*).

“*Being in this role now, I’m very passionate about blood donation and plasma donation, and make a real concerted effort, not just in my working career life but also in my private, to get the message out to the wider community and all my friends and everyone, and family. To talk about it as often as we can to make sure that we’re getting a good positive communication about how important it is to donate*.” (*Respondent 17, D-F*).

For some, their views on the importance of WOM stemmed from a better understanding of the importance of blood donations, in the context of treating various illnesses, such as knowing someone affected by leukemia or cancer who needed blood-derived products: “*I’m very passionately personally invested in the connection between treatment and blood donation*” (Respondent 13, D-F). This type of WOM motivation has also been identified in other blood donation studies focused on non-employee donors [53].

Respondents increased knowledge appeared to result in a greater sense of motivation to educate others about blood donations, including in relation to blood types, changes to eligibility criteria, and dispelling myths or misperceptions. The need to educate and share information was identified by participants in both D-F and O-F roles:

“*I think it’s important to expand people’s understanding of it’s not just whole blood, there’s platelets, plasma and all these other options as well.*” (*Respondent 1, O-F*).

“*Why I go and talk to people is I feel there are gaps where people aren’t getting that education and information that really – I don’t know – lets them know that they can donate.*” (*Respondent 3, D-F*).

### WOM opportunity in a dual role

The most common trigger for all participants to discuss blood donation was when they were asked about their work. Whether talking to a family member, friend or stranger, common questions like ‘what do you do for work’ or ‘how is work going’ enabled an easy transition into discussing blood donations.

“*When you say where you work, if it’s somebody that you haven’t met before, like a cab driver or a new social situation, it pretty much comes up.*” (*Respondent 2, O-F*).

One D-F participant proactively shared their roster with friends to encourage them to donate when they were working at the donation center. Other D-F participants were less enthusiastic about performing a phlebotomy on people they knew.

“*I have a few friends where I’ll send them my roster and I’ll be like, ‘You can always come in when I’m there and we’ll have a chat and I’ll do your needle and we’ll just hang out while you’re there’.” (Respondent 19, D-F)*.

*“Sometimes it’s a bit tricky to recruit people because you can end up being the one putting their needle in … If you’ve ever experienced the feeling of knowing that person and then missing their needle or having something go wrong, and then you’re associated with that forever and it can be a bit too much sometimes.”* (*Respondent 5, D-F*).

Some participants highlighted that when someone found out where they worked, this either resulted in a lot of questions or prompted them to justify why they had not donated blood.

“*The moment people find out where you work or what you do, then they have a lot of questions that they want to ask, and you can kind of fill those in*.” (*Respondent 4, O-F*).

“*When they find out where you work, you create automatically in the person asking the question the greatest guilt trip. It’s more so than pronouncing new year’s resolution about losing weight – ‘I’ve always wanted to donate but I’ve never done it’. It’s amazing the guilt association [among] people that haven’t donated*.” (*Respondent 11, O-F*).

Being questioned about work is a trigger unique to employee-donors. However, participants also highlighted that the post-donation bandage, blood type key chain, and personal schedule questions, also prompted conversations about blood donations. For some, donating blood was a normal activity they talked about, as with any personal activity.

“*When you talk about social scheduling and stuff. ‘Oh no, I’m off to donate blood. I’ll meet you in town afterwards.’ It opens it up*.” (*Respondent 2, O-F*).

*“People always ask, ‘Did you have a blood test? Are you okay?’ And it’s always easy to then spark up a conversation and say that ‘No, you’ve just gone and given blood’ and things like that. So it used to be something in my previous role that I used to do on a Friday morning before work, so I would still obviously still have bandages on my arm when I got to work.”* (*Respondent 18, D-F*).

While all participants shared examples of reactive WOM (e.g., responding to a question), there were more employee-donors in D-F roles (~ 50%) who shared examples of proactive WOM experiences compared with O-F roles (~ 20%). Many D-F employees indicated they were highly social people that loved to talk and thus share WOM. This is broadly consistent with other research on people who share WOM and eWOM in blood donation settings, as they like to share more broadly [10]. One of these participants moonlighted as a standup comedian and positively integrated blood donation WOM into their act – “*Hey, I’m a phlebotomist*” (Respondent 3, D-F) – to successfully recruit strangers. Another novel approach used by two of the participants, leveraging their unique experience at Lifeblood, was to comment on the suitability of someone’s veins for donating as an opening.

“You’re always assessing veins all the time. So it’s going out to the restaurant and I’m looking at whoever is serving the meal and I’m like, ‘*You’ve got really good veins. Have you donated before?’ And they look at you weird and I’m like, ‘It’s really a good cause’, and he’s like ‘No, I haven’t donated’. But I start the conversations that way sometimes.*” (*Respondent 22, D-F)*.

“*A lot of the times, well whenever you see a person with really good veins for example, I would always kind of like say ‘Oh, you’d be a good blood donor’. Just by looking at the size of their veins, as a compliment*.” (*Respondent 24, O-F*).

A similar amount of D-F and O-F employees shared examples of proactive eWOM by posting blood donation content to their personal social media profiles. Such content could include sharing existing Lifeblood posts or sharing their own donation activity, and posts may be motivated by reminders about upcoming appointments or texts telling people their blood has just been used.

“*When I donate, I usually try and do the photo, bit of the machine or something, and I keep it very simple. Just say ‘Donating today, donate if you can’.*” (*Respondent 8, O-F*).

“I do share different campaigns depending on where we’re at in terms of blood supply. I will sometimes also share when I have reached a milestone as well for me.” (*Respondent 18, D-F)*.

Two of the participants in D-F roles also noted that as staff they actively encouraged other donors to post about their donation activity online.

“*But when I have a donor come in, especially a new one or young one, I actually do encourage them to take a photo. But it depends whether I actually get a chance to talk to them, or interact with them, then I will recommend, ‘Oh actually, we have a sign out there, it’s perfect for an Instagram or Facebook photo’.” So, I just kind of recommend, and show it to them, and I say ‘If you want to take one while you’re sitting here, I can help’, things like that*.” (*Respondent 25, D-F*).

“*I go, ‘Hey, I’ve got an even better idea. How about when you come down and donate, you take a photo of yourself and you put it on your social media, and I’ll give you some info and you can push it out there on your socials and show everyone what a good thing you’re doing’. … They love it. They love getting the photo.*” (*Respondent 17, D-F*).

### WOM ability in a dual role

The dual role has been recognized as an advantage for employee-donors, as they are exposed to ‘insider knowledge’ that provides additional detail around the need for blood and different blood types, what happens to blood after a donation (e.g., processing, testing, product development, recipients), and eligibility criteria [42, 43]. In line with this, participants felt that having more information about the various aspects of blood donation made it easier to talk about.

“*I think it definitely does give us the upper hand, or an advantage in a sense, because we know a little bit more about the processes involved in blood donation from start to finish. As opposed to your regular donor, they don’t know too much into the details of what the journey of their donation takes. So, although it’s not essential for them to know more about it, I think it definitely gives the staff, or those coming from a background in Lifeblood, an advantage in passing on that information to those who are curious*.” (*Respondent 23, O-F)*.

“*I feel like having more knowledge about it makes you more comfortable to be able to speak about it I think*.” (*Respondent 19, D-F*).

“*But coming into the organization as well and not just being a donor, you see the background that goes into everything like in the medical services, but definitely being back in manufacturing [area where respondent worked]. We go down into the labs and you just see the blood being processed and everything that goes on and order fulfilment, and sending out blood orders for critical orders and things like that. That just adds more to what I can talk to people about and just let them know the things that goes on with your blood and what happens with it.”* (*Respondent 8, O-R*).

However, some participants perceived much higher expectations for them as employee-donors, to know more and be able to respond to specific questions, criticisms, and concerns regarding blood donation and Lifeblood processes. Many of the participants who had donated before joining Lifeblood noted that their conversations had shifted since adopting the dual role. This included questions around techniques to avoid having an adverse reaction, how to change contact preferences, where donation mobile units visit, what donation type is best for their blood type, and what happens to their blood donation. This indicates that those receiving WOM from employee-donors see them as organizational representatives, and thereby expect them to be able to respond to all their blood donation questions (irrespective of their role and/or training), more than just an average individual donor, who might not be expected to have this detailed knowledge.

“*I think they ask more questions around it because I do have more information around answers for them now*.” (*Respondent 15, D-F*).

“*People want to know – expect you to know everything about everything, the blood type stuff.*” (*Respondent 2, O-F*).

There were often questions and criticisms of blood donation eligibility criteria, which are constantly changing. Some participants had no difficulties responding to such feedback.

“*Yeah, a lot of questions about process, lot of questions about eligibility as well. That gets fired around a lot. ‘Why can’t I donate if this? Why can’t I donate if that?’ and that sort of stuff, and I think again now that you work here, you get that next level of knowledge where you can actually explain the reason why as opposed to it just being like, ‘I don’t know’*.” (*Respondent 21, O-F*).

“*I personally have got enough information, but to say that for everybody – because I’ve been here 40 years. For a new person that might be a bit more difficult and so they need pointing in the right direction to where they can gain all the information they need to help*.” (*Respondent 11, O-F*).

Other participants felt unsure about how to discuss the rationale for certain eligibility and deferral criteria.

“*I’m not as well-versed in all of the information. I know a fair bit because obviously I participate in donating, but I do find that people will ask very specific questions and I’ll have to refer them to the website for that*.” (*Respondent 1, O-F*).

“*I think blood donation is a really challenging conversation for people who don’t have experience, for people who don’t understand – or even people that do, it’s really complicated. And I think the easier that Lifeblood can make our staff, our 3,500 people understand those conversations and navigate those conversations with their friends and family in a really simple kind of way, will help us to be able to recruit our friends and families to donations without feeling like I did when I had that first post up – ‘Well I’m gay and you guys won’t let me’– you know that first smash and then it’s like you get gun shy and you’re like ‘Well I don’t know how to respond to that. What should I say and how should I deal with these things?’ So yeah, that for me has really curtailed my ability to be a really passionate advocate for blood donation.*” (*Respondent 13, D-F*).

While generic questions, such as how long donation takes and where they can go to donate are similar to those documented in other non-employee blood donor WOM research [29], receiving criticism of the policies related to who can donate blood appears unique to those in dual employee-donor roles, and has not been identified in past literature on donors.

## Discussion

This research explored the current practice, motivation, opportunity, and ability of WOM and eWOM among Lifeblood employees that also donate blood. There is clear alignment between individual and organizational values, with some blood donors seeking out employment at Lifeblood while others were motivated to start donating due to their employment. The lived experience of donating blood is considered beneficial not only for the employee-donor’s job (i.e., better understanding of what they are asking of donors), but also in appearing more authentic when talking about blood donation and encouraging others to donate. Yet despite the clear values alignment, being an advocate for blood donation is not generally considered a necessary part of the role as an employee-donor (excluding those employees in community outreach positions whose job is to advocate for blood donation), nor do they feel a responsibility to recruit others [33]. Although many participants voluntarily took on this advocate role, it is unclear if they would have been comfortable being asked to do so, as has also been suggested in other literature [38]. A common WOM and eWOM goal among employee-donors that participated in this study is to educate others about blood donation and different blood types, inform of changes to eligibility criteria, and generally dispel myths or misperceptions.

This research also examined whether the WOM experiences of these dual-role employee-donors differed, based on whether they were in D-F (e.g., donor center, marketing) or O-F roles (e.g., manufacturing, finance). For example, the participants in D-F roles reported feeling more aware of the importance of encouraging others to donate blood, often due to a better understanding of the various ways that blood donations are used to improve patient outcomes. Those in D-F roles were also more likely to be proactive in their WOM activity (e.g., bringing up the topic of blood donation in conversations with people without a trigger), actively seeking out and encouraging others to donate. Although participants in both D-F and O-F roles were equally likely to advocate blood donation online through unprompted positive eWOM. Any differences in unprompted positive eWOM were more to do with how they used social media platforms. Both roles were also just as likely to engage in reactive WOM and eWOM, triggered by events such as recent donations, reminders of appointments, or questions about their work or recent donation activity.

Among those participants that proactively and reactively engaged in WOM and eWOM about blood donation, it was suggested there were both advantages and disadvantages to having a dual role. For example, while being an employee-donor triggers more conversations about blood donation when asked about their work, this can lead to negative reactions that can be difficult for blood service employees to navigate. Some participants reported receiving guilt-induced rationales for why someone had not donated, or feedback on peoples’ past negative donation experiences, and concerns and criticisms of the eligibility criteria. The deterrent to sharing WOM of being confronted with these negative reactions has not been identified in previous research on donor WOM. Employee-donors often feel better equipped to answer questions and address negative feedback based on their knowledge [42, 43]. There are often higher expectations of employee-donors being able to explain and/or defend Lifeblood’s policies. Many participants in this study, however, did not feel fully equipped to respond to more complex questions, which suggests that additional internal training may make them more comfortable with sharing and directing to the relevant information. While not discussed in this study’s focus groups, there appears to be an added layer of complexity when encouraging employee-donors to engage in WOM and eWOM, in terms of the balance between representing the organization when answering questions (i.e., providing accurate, non-confidential information) while also drawing on and remaining authentic to their own lived experience.

From a practical perspective, Lifeblood and other blood services have a beneficial referral resource from using highly committed employees, who are also donors to share WOM. This is something that blood services can encourage and support to make easier for these employee-donors. However, it is important that these organizations are considerate in terms of the roles (i.e., sharing WOM) that employee-donors feel comfortable with. That is, while people may be committed to the organization and blood donation, they may not all want to be proactive advocates for blood donation. The organizations need to ensure that employee-donors can provide potential donors with clarification on issues or concerns, and easily organize appointments, as well as encourage WOM and eWOM. This would allow them to leverage their experiences as both donors and employees.

To assist employees in having more confidence with sharing WOM, services such as Lifeblood could provide (additional) organizational-wide training, to ensure that all staff understand a wider set of operational and procedural issues. In the context of blood donation, this may be particularly important when there are significant changes to the deferral criteria (such as the end of the vCJD deferral [54]) to ensure employees understand changing processes and procedures. There is of course some limitation as to how much can be undertaken across all functional roles, especially those not directly involved with recruitment or blood collection.

Organizations also need to be careful that they do not place pressure on employes to donate or share WOM. As was identified, some employees resent this, which could result in maladaptive behaviors [34]. It should be noted that for our employee-donors, some indicated that they felt that it would be inconsistent for them to work for Lifeblood and not donate. As not everyone is able to donate, there may be a need for initiatives to alleviate these feelings as well.

## Limitations

Within this research we focused on WOM of employees who are donors. Future research should examine how employees who are non-donors, lapsed donors or deferred donors share WOM, as they too will interact with the broader community. Given that they work for Lifeblood it would be anticipated they would be positive about their organization given its social mission and thus willing to share WOM, as it would come up in social settings discussing where people work. However, it is possible that these people may choose not to share WOM, as doing so could result in others enquiring on their donor status and then asking why they do not donate. In the case of deferred donors this might require them to feel a need to share personal information that they would prefer not to. While research on deferred donors and WOM is sparse, the results suggest that those deferred do also share positive WOM [55]. However, it should be noted that this work did not include employees who were deferred.

Lifeblood does not collect information on the blood donation status of employees and thus it is impossible to identify the composition of this population to comment on how representative or not our participants were. Further, while our participants were employed in a wide range of roles from across Australia, we did not specifically investigate whether their experiences varied by the rural or urban setting that participants worked and/or lived in. As engagement in WOM may be affected by the opportunities that exist to engage in the behavior talked about, and in the context of blood donation in Australia, these may be comparatively fewer in rural areas, additional research should explore the impact of urban or rural settings on whether and how people engage in WOM about blood donation.

## Conclusion

Being an employee-donor is a double-edged sword. WOM often stems from being able to share authentic lived experiences of donating blood, but with the expectation that as employees they will know more than a typical donor and have organizational and blood donation expertise (irrespective of their position or training). While being a staff member provides more opportunities and triggers for proactive and reactive WOM, many employee-donors also perceive additional barriers. There is often a lack of clarity on how they should appropriately represent the organization (i.e., Lifeblood) and how they can navigate the challenging nature of the types of questions that recipients feel appropriate to ask them. These are not barriers faced by WOM donors who are not employees. More research is needed in relation to those in dual employee-customer roles within healthcare or non-profits, to determine whether these barriers are issues faced more broadly, and how employee-customers can be most effectively supported to engage in positive WOM.

### Electronic supplementary material

Below is the link to the electronic supplementary material.

Supplementary Material 1

## Data Availability

The qualitative dataset analyzed for this study cannot be shared openly to protect study participant privacy, and consent was not provided by participants for their data to be shared publicly.

## Footnotes

1 Information Age, gender and COB data for blood donors were extracted from the Lifeblood National Blood Donor Management System by Lifeblood and provided to the researchers by Lifeblood. Demographics on the demographics of Lifeblood employees is drawn from https://www.lifeblood.com.au/sites/default/files/resource-library/2023-11/lifeblood-annual-report-2022-23-7.0-FA.pdf51. LifeBlood: **Australian Red Cross Lifeblood 2022–2023 Annual Report**. In.: LifeBlood; 2023. (accessed 01/05/2024) [51].

## Abbreviations

WOM: Word-of-mouth
eWOM: Electronic word-of-mouth
MOA: Motivation, Opportunity, and Ability framework
COM-B: Capabilities, Opportunity, and Motivation – Behavior framework
R&D: Research and Development
D-F: Donor-facing
O-F: Organization-facing
