# Supplementary material for: Word-of-mouth among blood service employees who also donate blood: a qualitative investigation of advantages and challenges for dual-role donors
Source: BMC Health Serv Res. 2024 Jun 14;24:736. doi: 10.1186/s12913-024-11181-y (PMC11179336; doi:10.1186/s12913-024-11181-y)
Supplement: Supplementary file 1 — Supplementary Material 1 [file 12913_2024_11181_MOESM1_ESM.docx]

SCREENING QUESTIONNAIRE

# DONORS-RECRUIT-DONORS (DB20-01): STUDY 1A STAFF

**Are you a Lifeblood member of staff who also donates blood, plasma, or platelets?**

**Have you ever talked about what it’s like to donate or to be a donor with a non-donor?**

**Would you be willing to help Donor Research with a new project exploring “word-of-mouth” and recruitment?**

If this is you, we’d love to talk to you in some small group MS Teams discussions with other Lifeblood staff members who are donors about your word-of-mouth experiences before we go out and talk to non-staff donors. We believe our fellow Lifeblood staff members might have some unique perspectives on what it’s like talking to non-donors about donating.

**If you’re interested in participating please fill out this short survey so we can find out a little bit about you, and so we can get in touch when the time is right.**

Your responses will only be used by the research team to ensure a broad representation of donors with different backgrounds and donation experience. We are only running five sessions, so spaces are limited. Priority will be given to those who respond first and represent cohorts that are less common.

| 1. What is your name? | *[open-ended]* |
| --- | --- |
| 1. What is your age (in years)? | *[open-ended]* |
| 1. How do you describe your gender? | - *[open-ended]* - Prefer not to say |
| 1. Where were you born? | - In Australia - In another country |
| 1. Please choose your ancestry from the following groups: | - European   - British/ Irish   - Western European   - Eastern European   - Southern/ Mediterranean European   - Scandinavian - East and South East Asian   - Burmese   - Chinese (e.g., Han Chinese, Indigenous Taiwanese, Other Chinese Asian)   - Filipino/ Pilipino   - Javanese (Indonesia)   - Japanese   - Khmer   - Korean   - Lao   - Malay   - Sundanese (Indonesia)   - Thai   - Vietnamese   - Other South East Asian - Central and Southern Asian   - Central Asian (e.g., Afghan, Uzbek)   - An Indian Ethnicity   - A Pakistani Ethnicity   - Sinhalese   - Tamil   - Other Southern Asian (e.g., Bengali, Bhutanese, Nepali, Tibetan) - Middle Eastern/ West Asian   - Arab   - Assyrian   - Ashkenazi Jewish   - Middle Eastern Jewish   - Kurdish   - Persian   - Turk   - Lebanese   - Other Middle Eastern/ West Asian - Oceania/ Pasifika   - Aboriginal Australian   - Torres Strait Islander   - Micronesian (e.g., Chuukese, Chamorrow, Marshallese, Nauran, Palauan)   - Melanesian (e.g., Itaukei (Fijian), Indo-Fijian, Papuan, Solomon Islander, ni-Vanuatu)   - Polynesian (e.g., Maori, Samoan, Tongon, Hawaiian, Tahitian, Cook Islander) - African   - Afrikaner   - North African   - Central and Western Africa   - Southern and Eastern Africa (not including Afrikaner) - Americas   - African American (North, South, or Central America)   - Central American   - Hispanic, Latin, or Portuguese American   - First Nation or Native Norm American (e.g., Inuit, Iroquois, Sioux)   - Indigenous Central or South American (e.g., Makuxi, Ticuna, Mapuche, Quechua) - I’d prefer not to say |
| 1. When did you last attend a donation appointment? | - October 2021 - September 2021 - August 2021 - July 2021 - June 2021 - May 2021 - April 2021 - March 2021 - February 2021 - January 2021 - December 2020 - November 2020 - October 2020 - Over 12 months ago |
| 1. How many times have you donated whole blood, plasma and/or platelets with Lifeblood? | *[open-ended]* |
| 1. How many years have you worked at Lifeblood? | *[open-ended]* |
| 1. What division of Lifeblood do you currently work in? | - Business Growth and Innovation - Clinical Services and Research - Corporate - Corporate Strategy and Transformation - Donor Services - Finance - Information and Communications Technology - Manufacturing and Quality - People and Culture |
| 1. When would suit you most to attend a focus group? *Select all that apply.*   *Note:* Participating during work hours is dependent on approval from your line manager. | - Weekdays (morning) - Weekdays (afternoon) - Weekdays (evening – after 5pm) - Saturday (daytime) - Sunday (daytime) |

**FOCUS GROUP QUESTION GUIDE**

**INTRODUCTION**

Thank you all for taking the time today to participate in our focus group.

As a staff member of Lifeblood who also donates, we are interested in learning about your perspectives and experiences (or lack thereof) of talking about and promoting blood donation to others.

During the session, we may refer to this as word-of-mouth, which can include donors saying positive or negative things about their blood donation experience or Lifeblood as an organisation, talking or posting on social media about blood donation generally, or recommending others to donate blood.

**GETTING TO KNOW YOU** (~20mins)

**To start, could everyone please tell me a little about what you do and how long you’ve been at Lifeblood, as well as when and why you started donating blood?**

1. How long have you worked at Lifeblood? In the same division as you are now?
2. Have you donated prior to working at Lifeblood?
   - *If yes, what got you interested?*
   - *If no, why did you start? Was working at Lifeblood a motivator for you or was it a coincidence of timing?*
3. What type(s) of donation do you usually make (whole blood, plasma, platelets)?
4. **Do you like to donate?**
   - *Why/ why not?*

**WOM 1 – CURRENT PRACTICE AND PRIOR EXPERIENCE (~30 mins)**

**Before you started donating, did others ever talk to you about blood donation, and if yes, what sorts of things did they say and how did it impact your decision to donate?**

1. Did you ever hear word-of-mouth about blood donation prior to becoming a donor?
   - *What sort of things did you hear?*
   - *Who did you hear it from?*
   - *What did you think about those things?*
   - *How did it impact your decision to donate?*

**Now that you have donated, can you tell me about the times that you’ve spoken to others about donating blood, what prompted these conversations and what did you speak about?**

1. Have you ever started talking to others, especially non-donors, about donating blood?
   - *Or do others tend to start these discussions with you?*
   - *Who do you have these discussions with?*
   - *How do these conversations start?*
   - *When people learn you work at Lifeblood, is this a prompt to start talking about donation?*
2. What sort of things do you focus on when talking about blood donation?
   - *Your experiences? Positive or negative?*
   - *“Have you ever fainted?”*
   - *People’s fears about donating (e.g., needles)*
   - *Broader barriers to donating (e.g., convenience, previous deferrals)*
   - *Cultural or religious barriers*
   - *People’s lay beliefs about donating (e.g., donating blood makes you weak)*
   - *Refreshments?*
   - *The warm glow?*
   - *The process?*
   - *The convenience?*

**For those of you who had donated before working at Lifeblood, do you think the nature of what you talk about has changed, and is it easier or harder to talk to others now that you work at Lifeblood?**

1. Do you think the topics discussed are different when conversations are prompted by you being a Lifeblood employee or donor?
   - *Broader policies about blood donation (e.g., MSM deferral, Mad Cow deferrals)?*
   - *Mechanics of donating? How to get started? The actual process?*
   - *Is it easier or harder to talk about blood donation as a donor or staff member, have conversations changed, confidence?*

**A lot of what you have mentioned has been more face-to-face conversations, what about online activity such as on social media?**

1. When or where do these conversations usually happen?
   - *Online or in-person?*
   - *Social events like dinner parties or barbecues?*
   - *School pick-up or when chatting with other parents?*
   - *At work? Have you ever talked to another Lifeblood employee about being a donor?*
   - *Anywhere else? (e.g., hairdressers)*

**How do people usually respond? Have you ever had negative responses or had to deal with negative feedback, and how did you manage this?**

1. For those who have started conversations or posted on social media about donating blood, how do people respond?
   - *Positive or negative?*
   - *Interested or uninterested?*
2. Do you think you ***have ever “convinced” someone to donate or seek out more information about donating?***
   - *If yes or no,* ***how did this make you feel****?*
   - *More or less motivated to engage in word-of-mouth and/ blood donation?*

***Do you see your role as a Lifeblood staff member, donor and advocate as similar or different? In what way? Do you see promoting blood donation as part of your role as a donor? Why/ why not?***

1. Can you tell us a little bit about your motivation (or lack of motivation) to talk to others about donating?
   - *How do you think your motivation compares to the motivation of donors who do not work for Lifeblood?*
   - *What makes it difficult to talk to others about donating (barriers)?*

**WOM 4 – LIFEBLOOD SUPPORT (~20 mins)**

**Finally, what could Lifeblood do to support word-of-mouth activities by donors? Or help make these conversations with potential donors more effective?**

1. What do you think Lifeblood could do to support these conversations for donors more generally?
   - *Such as produce a guide or fact sheet*
   - *Conversation starters*
   - *Content to share on social media*
2. Is there anything else you would like to add?
